# Immersive, Open-World Virtual Reality for Dementia Care: NeuroVRX Pilot Study

**DOI:** 10.3390/jcm14238465

**Published:** 2025-11-28

**Authors:** Martin Eckert, Thomas Ostermann, Jan Peter Ehlers, Gregor Hohenberg

**Affiliations:** 1 Stabsstelle für Digitalisierung und Wissensmanagement, Hochschule Hamm-Lippstadt, 59063 Hamm, Germany; gregor.hohenberg@hshl.de; 2 Fakultät für Gesundheit, Universität Witten-Herdecke, 58455 Witten, Germany; thomas.ostermann@uni-wh.de (T.O.); jan.ehlers@uni-wh.de (J.P.E.)

**Keywords:** medicine, cognitive impairment, dementia, Alzheimer’s disease, video games, serious games, exergames, virtual reality, open-world

## Abstract

**Background**: The increasing prevalence of Alzheimer’s disease and related dementia are a global problem generating social and economic burdens. Cognitive Stimulation Therapy is a non-pharmaceutical aid for people with dementia. In this context, digital and virtual reality approaches are underinvestigated, especially with respect to explorable open-world environments. The pilot aims to evaluate the feasibility, acceptability, and safety of an immersive, open-world virtual reality application for people with dementia. **Methods**: We conducted a single-arm, unrandomised study with three male participants diagnosed with dementia. The intervention consisted of a single virtual reality session in an immersive, open-world environment, where participants were able to explore freely while seated, using arm movements and head control to navigate an avatar. **Results**: All three participants finished the session without the occurrence of adverse events. The mean session time was 28 min, and the average walking distance was 0.9 km, with 1210 steps on average. Questionnaire results indicate acceptance and a positive attitude toward the usability of the intervention. We measured minimal changes in mood. Anecdotal reports indicate high immersion and autobiographical stimulation. We detected no adverse events or occurrences of cybersickness. **Conclusions**: Immersive, open-world virtual reality proved to be feasible, safe, and well accepted by the participants. The combination of state-of-the-art hardware and exploration-based software design enabled cognitive and motoric stimulation. The results indicate strong feasibility for the application of exploratory three dimensional virtual reality applications and further support the execution of controlled trials to assess therapeutic outcomes.

## 1. Introduction

Alzheimer’s disease and related dementia (ADRD) are a considerable challenge not limited to Western countries. In addition to personal and social challenges, the cost of care and treatment for persons with ADRD poses a problem for the healthcare sector. The cost of dementia care and treatment alone is estimated at USD 23,796 person with dementia globally on average, amounting to USD 1313.4 billion for 55 million people in 2019, as calculated by Wimo et al. [1], demonstrating consistency with recent publications of global dementia burden calculations—see Zhong et al. [2] and Zhang et al. [3].

### 1.1. Background and Motivation

The most recent World Health Organization (WHO) report, which was based on Global Burden of Disease (GBD) 2021 data [4], describes an increase in dementia. This report estimates that 55.2 million people were living with dementia in 2021, a number projected to reach up to 78 million in 2030 and 139 million in 2050 [5]. Multiple factors like cardiovascular disease, high body mass index (BMI), and tobacco use contribute to Alzheimer’s Disease and Related Dementias (ADRD) [6,7,8,9]. An option to counter this development is Cognitive Stimulation Therapy (CST). CST is a form of psychological treatment for people with mild to moderate ADRD symptoms. In 2014, the World Alzheimer’s Report recommended that CST is offered to people with dementia whenever possible [10]. It is a non-pharmaceutical approach, aiming to stimulate cognitive domains such as memory, attention, language, problem-solving, and decision making.

A large-scale review from the Cochrane Library in 2023 that included 2704 participants, of whom 1432 received cognitive stimulation, found cognitive stimulation in ADRD clinically important [11]. Noticeably, only 2 of 300 screened studies used digital technology to deliver cognitive stimulation [11]. More recent reviews and trials are demonstrating a interest in electronic serious games and Virtual Reality (VR)-based cognitive applications for people with ADRD’s; however, the majority maintain a structured and task-oriented design, with a focus on scores and timing rather than immersive free exploration. See Chang et al. [12], Navarro-Ramos et al. [13], Jeong et al. [14], and Yu et al. [15].

Exploring and discovering are inherent to humans and crucial for children’s cognitive development; see Poli et al. [16]. This behaviour is associated with a reward functionality that is linked to dopamine receptors of the brain. Stimulation of the nucleus accumbens reinforces exploratory behaviour by assigning rewards to new information and creates a motivation to discover, as modelled by Kakade et al. [17]. We find examples of the “fearless explorer” not only in history and literature but also in science, as demonstrated by the work of Liu et. al. [18]. They were able to correlate visual exploration with increased hippocampal activity, suggesting that the hippocampus plays a significant role in binding information during exploratory behaviour, see Liu et al. [18]. Not all People with Dementia (PwD) have the option to explore and discover the world as they did before their diagnosis. Cognitive impairments, such as memory loss and diminished judgment and decision-making, create safety concerns. It is known that individuals with ADRD can wander without a destination, getting lost and creating environmental problems and even accidents; see Banerjee [19] and Agrawal et al. [20]. Social stigma and demoralisation often reduce confidence and result in withdrawal from activities, as found by Brookman [21] and Ono et al. [22]. Recent surveys indicate widespread negative attitudes and fear of discrimination; see the World Alzheimer’s report 2024 [23]. Caregivers often impose well-intended restrictions to ensure safety but limit autonomy and opportunities to discover.

One safe way to explore is to use video games combined with VR. This new technology enables its users to experience an immersive experience with the opportunity to promote cognitive flexibility and provide stimulation through engagement and escapism. The involvement of visuospatial processing, motor coordination, and decision-making engages cognitive and sensory pathways of the brain with a potential influence on mood, stress, and emotional regulation and well-being. Software is often found to be non-immersive and outdated in terms of graphical representation; see our prior work [24]. Sea Hero Quest, a mobile app that garnered significant attention as a serious game in 2019, was primarily designed for data collection and limited immersiveness [25]; see the also the follow-up from Spiers et al. [26]. The design of serious games often evolves around training tasks like memory, requiring time or score challenges. The motivation of this work is to minimise traditional game mechanics and promote player agency to encourage exploration driven by intrinsic motivation to discover rather than collect rewards.

### 1.2. Research Objective

The goal of the Neurocognitive virtual reality exploration (NeuroVRX)Pilot Study is to develop and evaluate an interactive virtual reality system combining physical and cognitive exercises to generate cognitive stimulation and improve well-being in older adults with early to mild ADRD. The design of the VR application is centred around realistic, free exploration and high immersion in the VR.

This research sought to determine if exposure to the virtual reality is accepted by the PwD and how they perceive the immersive user experience and game mechanics. The research also considered the potential occurrence of simulator sickness and changes in the subject’s mood and engagement levels.

## 2. Methods

### 2.1. Research Design

The study was designed as a single-arm, non-randomised pilot study. No control group was included. This study was designed to explore feasibility, acceptance, and safety to provide information for future trials. Ethical approval was obtained beforehand, and informed consent and GDPR statements were obtained from all participants. To gain initial proof of concept and obtain real-world data, we decided to conduct this early-stage pilot with a single-arm study design and a small-batch (*n* = 3) sample size. We did not aim to demonstrate efficacy in this early stage of research.

The sessions were standardised for safety. To mitigate risks of falling, especially among older adults, the session was conducted in a sitting position with the subject using their arms movement. Earlier tests during development indicated balance issues, even with healthy participants; therefore, we opted to design a seated experience.

The seated position maintains the exercise factor of the prototype while minimising the risk of falling during the VR session. During active sessions, the participant was observed by study personnel in real time. If discomfort arose, it was determined whether the participant wanted to continue or discontinue the VR experience. Immediately before and after the session, a safety check was conducted to guarantee comfort and safety. In case of adverse events, such as dizziness, discomfort, or anxiety, the session was paused, and the safety of the participant was assured. The session was then resumed or aborted. If circumstances were stable, the CSQ questionnaire was completed, even in the case of an aborted session. A short onboarding phase helped identify problems with the VR setup before the start of the session. Telemetry data were recorded continuously by the software (session time, distance, and steps). Standardised questionnaires were applied to measure mood, acceptance, usability, and cybersickness. During and after the trial, the data were stored in compliance with the General Data Protection Regulation (GDPR).

### 2.2. Participants

Participants were identified and recruited with the help of local dementia networks. Their eligibility was assessed before the VR session and according to defined criteria; see Table 1. The participants were recruited based on existing diagnostic assessments. No additional diagnostic tests were conducted in the screening period or during the execution of the NeuroVRX study.

Informed consent was provided by each participant in the presence of a caretaker prior to the VR activity. PwD with a history of epilepsy, anxiety disorders, or impaired hearing or vision were excluded. We also excluded PwD with advanced dementia symptoms because they did not understand the activity.

### 2.3. System and Application Design

The system design integrated high-end consumer hardware (Meta Quest 3, Meta Platforms, Inc., Menlo Park, CA, USA) with a consumer gaming laptop. A detailed list of the hardware is provided in Appendix A, Table A1. For performance reasons, a dedicated WiFi Router enabled a wireless setup; for details, see Appendix A, Table A2. The modular software stack ensured stable performance and immersive interaction in the VR. It was created on top of an open-world three dimensional (3D) role-playing environment; details of the software stack are listed in Table A3. The participants were free to explore the vast, detailed environment. Meta Quest 3 controllers were used to facilitate natural movement and enhance immersiveness as much as the seated position would allow.

The environment features high-resolution graphic details with dynamic light processing and real-time interaction with the environment. During the development phase, one of the development goals was to keep the frame rate above 120 frames per second, as there is scientific evidence of a threshold for lowering discomfort and visual stress; see Wang et al. [27]. The challenge was to balance visual appearance and calculated frames to create a suitable and immersive virtual environment. Highlights include reflections of the sun or moon in water and flickering fires. The audiovisual simulation features a spatial audio environment that is synchronous with the video environment. Examples of the sounds are rain falling on the ground while thunder rumbles in the distance, footsteps audible on different surfaces, a campfire crackling, and waterfalls rushing down cliffs. Animals and people can be located based only on the sounds they make.

To enable physiological activation, the participant’s view is controlled by using the head to create an immersive feeling and activating the neck and shoulder muscles. Although the setup would allow for walking and standing interaction, the avatar is controlled by holding two handheld Meta Quest 3 controllers and imitating a walking movement with both arms while maintaining a safe sitting position. To ensure emotional and physical safety, the following adjustments were made to the virtual world: falling, which can occur while climbing, does not have any severe effects on the avatar. Natural, aggressive animal behaviour has been suppressed, though the option to interact with the animals (e.g., petting) is maintained. Violence and territorial behaviour by virtual citizens have been suppressed to avoid aggression and conflict.

In accordance with existing legal frameworks, interested readers of this publication are advised to direct any further requests for information on implementation and reproducibility to the authors.

### 2.4. Outcome Measures

This section describes the variables, questionnaires, and measurement tools used to address our research questions. The questionnaires were designed to collect qualitative and quantitative information about the experience of the activity. Four questionnaires are answered during one session. The questions were read out loud by the study personnel, and the participants provided their responses vocally in the presence of a caretaker.

The first questionnaire gathered demographic data and background information of the participant, which was necessary to provide context on gender, age, and familiarity with digital games while maintaining anonymity.

Afterward, the Aktuelle Stimmungsskala (ASTS) questionnaire was applied to determine immediate pre- and post-mood profiles of the subject. This questionnaire addresses the main research question directly. We applied the German short version of the Profile of Mood States (POMS)—see McNair et al. [28]—to this task; see also Dalbert et al. [29]. The questionnaire contains nineteen items grouped into five corresponding mood states (Sadness, Hopelessness, Tiredness, Anger, and Positive Mood) evaluated using a five-point Likert scale. The internal consistency of the test has been proven to be very reliable (Cronbach’s Alpha values of 0.83 to 0.94).

The Cyber Sickness Questionnaire (CSQ) was applied after the activity in the adapted VR version (CSQ-VR). This questionnaire has six items and three subscales (nausea, vestibular, and oculomotor), with two items for each subscale. The overall consistency score reported by Kourtesis et al. [30] was measured with *n* = 39 participants exposed to three VR rides with linear and angular accelerations. The total Cronbach’s Alpha score is reported to be 0.865. For comprehension, the questionnaire was translated and verified by the author. Three categories (nausea, vestibular, and oculomotor) were evaluated using a seven-point Likert scale. Two questions in each category are summarised, and the three categories are summed to produce the total score.

User experience and mechanics were assessed using the Virtual Reality Neuroscience Questionnaire (VRNQ). The test was created and validated by Kourtesis et al. [31] with *n* = 40 participants and three VR sessions per participant (120 total datapoints). The original questionnaire features 20 items in four categories (user experience, game mechanics, in-game assistance, and VR-induced symptoms and effects). Regarding consistency, a Cronbach’s Alpha value in the range 0.89–0.90 is reported for all domains, indicating good to excellent consistency. For this study, the category in-game assistance was removed from the questionnaire, since no in-game assistance is provided in our designed experience. The category VR-induced symptoms and effects was also dropped and replaced by the newer CSQ, which is described above. Further the question about in-game encounters has been removed, due to the fact that we did not want to overwhelm the participants at the first VR interaction. The scores have been adjusted accordingly.

Two remaining categories (user experience and game mechanics) were captured using a 7-point Likert scale, with five questions for user experience and four for game mechanics. The fifth social interaction question from the game mechanics category was removed because social interaction does not occur in the initial session design. Evaluation scores were adjusted accordingly. The categories build a sub-score with a threshold value, and the category scores can be summarised. An overview of the variables is provided in Appendix A, Table A4. The original questionnaire was been translated into German, and changes and adjustments were approved by the original author. The questionnaires can be found in the OSF repository; see https://osf.io/r6up9, accessed on 20 November 2025.

## 3. Results

### 3.1. Participant and Session Characteristics

In total, three participants were recruited for this study. The participants are male, and the average age was 77 years, with two PwD over 80 years of age. None of the three were prone to travel sickness or motion sickness, and none had experience with video games or specific VR applications.

After explaining the trial and clarifying the risks, the informed consent form and data privacy agreement were signed in the presence of a caretaker. First, the participants’ demographic data and the ASTS pre-questionnaire were filled out. Following fitting of the VR headset and explanation of the controls, the session began. All three participants started exploring at the same forest location within the application. Subsequent to the end of the session, study personnel checked if the participant was suffering any type of disorientation or sickness to ensure their safety. Then, the questionnaires were administered in the following order: ASTS-post, VRNQ and CSQ-VR.

### 3.2. Session Results

All session data were collected in the forest scenario. To keep the results comparable, the pilot study introduced the forest setting only, even though there is considerably more potential for exploration in the 37 m^2^ VR. Table 2 provides a comparison of the VR sessions with three different participants in terms of duration, distance, and steps taken.

There was no limit to the length of a single session. The sessions averaged 28 min and were terminated by the caretakers, who were present during the session, in the case of Participants B and C due to notable fatigue. Participant A terminated the session vocally by himself. The participants walked an average of 800 m and took 1200 steps on average. Participant B was immersed in the VR for as long as 34 min, but the farthest distance was covered by Participant C, who took fewer steps. The step size depends on the length of the arm swing movement, and it is possible to swing less or more. This movement transfers directly to larger or smaller steps. It was also observed that with a longer session time, the step size was reduced.

The three participants had no suggestions or remarks regarding the session, VR experience, or the content. All three participants would recommend the experience to other PwD.

### 3.3. Session Reports

#### 3.3.1. Session Report: Participant A

Participant A is an 81-year-old male who resides in a nursing home. We start to read through the informational material and informed consent and data privacy statements in the presence of staff. We chat casually while the system is set up in the participant’s room. We then fill out the questionnaires to start the actual session. He takes a seat on a swivel chair from the staff office in the middle of his room. Soon, he finds himself in a forest setting. Large pine trees reach up high into a clear blue sky. The sound of a chickadee is audible. The participant starts to move his arms in the thought-walking manner. His avatar takes its first steps towards stonier terrain. He notices a snowy patch and starts to move towards a snow field. Climbable rocks break through the field. Still walking through the field, his gaze lands upon one of the larger rocks uphill. In a very committed and engaged tone, he audibly says, “Ich will da rauf!” (“I want to climb up there!”). He decides to climb it. Checking out the right side of the stone, he finds it was too steep to climb. His avatar continues walking against the rock. He is walking around to find a shallower flank, and he starts climbing up the stone, step by step. Standing on top, the participant seems very proud of himself. He launches into an explanation of how he travelled the world when he was younger and starts telling a story. Then, he continues to explore the VR. During the session, he seems very committed and determined while maintaining a neutral emotional tone. It is necessary to remind him twice during the session that movement is achieved by moving the arms. In these instances, he stops his avatar and looks around for longer than usual, indicating a short-term loss of the movement he was engaging in earlier. On another occasion, he is so immersed that he wants to walk, standing up from his seat and actually using his legs to walk instead of performing the designed arm movement. This happens during a more intense balancing–climbing situation in the VR experience. Eventually, he becomes tired and wants to quit after 21 min. He asks to complete the session. Immediately after the session, he is asked if he feels any effects of motion sickness or disorientation. The participant denies feeling any effects, and we move to finish the questionnaires and end the session. While filling out the questionnaire forms, he remarks “yes, an interesting Experience” (“Ja, das war eine interessante Erfahrung”).

#### 3.3.2. Session Report: Participant B

Participant B is a 89-year-old community-dwelling PwD that lives at home with his wife. It is a warm summer day with high temperatures. After a short introduction by his wife, he sits on the couch, seemingly waiting absently. The laptop and the WiFi router are set up in the kitchen. He stands to get his glasses, and we sit down at the dinner table to talk about the trial and the session and to sign the paperwork. After, we both walk over to the chair, which is placed in the middle of the living room, and start fitting the gear while explaining the controls. Because there was no swivel chair available in the house, we decide to manually turn the chair during the session to help with the turning motion of the avatar. The participant puts on the VR rig (notably, it is not a problem to wear the glasses under the VR rig). After a short explanation, he starts in the forest area, just like participant A. Instead of walking uphill, he is intrigued by the green forest and starts to follow a path downhill through the forest. A brief encounter with a small fox crossing the road is accompanied by a laugh from the participant. After a brisk walk, he finds himself on the shores of a large lake. “Das sieht aber kalt aus, da geh ich nicht rein” (“this looks cold, i am not going to go in”) he states, continuing to walk beside the lake. The change in direction requires a manual shift of the chair that needs to be performed by an external person. After continuing the walk beside the lake, without further comment, he seems to change his mind and dips in. Both his wife and I stand in the adjacent kitchen room and watch his stream on the laptop. She tells me that he was a regular swimmer until he could physically no longer swim—he went swimming at the pool every morning. He continues to cross the lake, sometimes diving under the water and watching the lakebed scenery. The participant continues to really enjoy the water. He finishes crossing the lake, continuing to explore the forest on the opposite side. Eventually, he becomes tired, and his steps become less enthusiastic after more than 30 min. In an agreement with the caretaker (his wife), we decide to end the session. I leave after finishing the questionnaires and disassembling the setup.

#### 3.3.3. Session Report: Participant C

Participant C, who is significantly younger, is met at the VR healthcare laboratory of the Hochschule Hamm-Lippstadt. After completing an introduction and the paperwork, he quickly sits down in the centre of the room and starts to explore uphill, toward the snowy patches, and continues on to discover a stony ridge. Soon, he discovers a hole in the ridge, which potentially leads somewhere. He says, “Oh da oben, da kann man bestimmt rein” (“I could go up and in there”). He starts to explore a dimly lit rock cave with occasional sunlight streaming in through holes above his head. It seems like the details of the immersive VR are soaking in; he takes his time to explore slowly, examining the detailed cave very closely. He climbs up steep ascents and walks down them without hesitation. The exploration continues farther until he seems satisfied and/or lost and wants to return outside. There is a loss of orientation since the cave is not modelled like a single room but features multiple pathways to be explored. On the way out, the terrain requires climbing a narrow pathway which is partly covered by large tree roots. The roots get in the avatar’s way, and the participant becomes stuck, needing assistance to wiggle the avatar free from the grasp of the roots. With guidance from the instructor, the avatar is freed, and the cave’s exit is found. Outside of the cave, under blue skies, the exploration continues for a couple more minutes. It seems that this adventure took a toll on his endurance and focus, as stated by the caretaker and not by the participant. The session ends about five minutes after leaving the cave. Even with such an immersive event, the participant does not seem confused and can clearly differentiate between reality and immersive VR. Paperwork is completed to end the session. He remarks, “Good 3D Recordings, Interesting” (“Gute 3D Aufnahmen, interessant”).

### 3.4. Application Mechanics and User Experience

The mean VRNQ-DE score is 46. This is above the threshold score of 45 to identify a suitable user experience and game mechanics. The mean values of User Experience and Game Mechanics were both above the individual category thresholds; see the results in Table 3. Note that one item from the Game Mechanics subscale was removed, reducing the score and related threshold by five points in the game mechanics category. For details, see Section 2.4.

Consequently, graphics, VR technology, and safety received high ratings. The categories immersion, pleasantness, orientation and exploration were reported with greater variability.

### 3.5. Changes in Mood

The results of the ASTS test were ambiguous. Only small differences were detectable, and *n* = 3 is not a statistically significant number with which to deduce a unique outcome. However the experiences described in the Session Reports are reflected in the results; see Table 4.

The ASTS score of Participant A on the subscale Positive Mood improved by three points. His overall behaviour during and after the VR session was more energetic than before. Participant B was observed laughing and enjoying himself while interacting with the VR activity; however, he did not report any of those emotions during the ASTS-post. Participant C, who decided to dive into the cave exploration, reported tiredness after the session, which was also measured by the ASTS test. Detailed scores can be reviewed in the appendix, see Table A5.

### 3.6. Cyber Sickness and Physical Effects

The CSQ-VR score was six for all three participants. A score of 6 is the lowest possible score; there were no occurrences of cybersickness during the sessions. In conclusion, there is a strong indication that modern, state-of-the art hardware and software enable the use of VR systems in elderly care with limited risk of cybersickness or other unwanted physical effects, like those measured in the CSQ-VR test; see Appendix A for the results in Table A6.

## 4. Discussion

The results of this pilot study indicate that immersive, open-world VR experiences are well accepted by individuals with early to mild dementia. The use of modern consumer-grade hardware and exploration-driven design to support immersiveness and therefore neurocognitive and physical stimulation is viable. Due to the design of the trial with *n* = 3 participants, no statistical inference was performed. No cases of cybersickness or disorientation were observed.

### 4.1. Interpretation of Results

The overall acceptance was high. All participants completed the full session with no negative feedback or aversions to the experience. The mean VRNQ-DE score (mean = 46) succeeded the threshold value (45).

The good results in terms of graphics and safety are notable, supporting the argument that high-quality hardware and software are required for a satisfying and safe experience. The included participants finished the VR sessions without symptoms of cybersickness, supporting the suitability of modern VR setups for early-stage ADRD applications. The duration of a single session (mean = 28 min) of dedicated physical activity (mean steps = 1200) suggests the potential to use VR as a physically engaging therapeutic tool. The different locations used during the study support a flexible mobile hardware setup that can be used at home, in residential care, or in therapeutic and institutional settings.

### 4.2. Limitations

This study is limited in its generalisability. The small sample size means that the results should be interpreted as an indication rather than conclusive results. The study design is missing a control group to compare the results to other non-VR experiences; further, the results need to be interpreted carefully as there may have been a novelty effect, as none of the three participants had any experience with VR. The use of a non-swivel chair for Participant B may have influenced the quality of the experience. As all participants recruited were elderly males, there are gender-related engagement and usability questions left unexplored, which limit the generalisability of this study. The ASTS mood results remain unclear due to the small sample size and single-session design.

### 4.3. Practical Implications

The acceptance rate and user feedback indicate that VR exploration experiences can be introduced in therapeutic and home environments with early-stage PwD. Seated positioning and engagement through walking-arm gestures are intuitive and safe. Minimal amounts of introduction and training are required before the session, making the setup very accessible to PwD with no prior gaming or VR experience. The explorative open-world design fosters decision-making and intrinsic motivation. This study offers a new paradigm for cognitive stimulation, contrasting with the task-oriented or reward-based design of serious games. It is possible that VR systems could become a non-pharmaceutical adjunct to CST or an engagement tool for therapists.

## 5. Conclusions

Overall, the immersive, open-world VR with explorative elements proved feasible and well accepted without adverse effects. Controlled trials are required to confirm the therapeutic value of this approach and means of integrating it into CST.

### 5.1. Summary of Key Findings

All three PwD completed the sessions without adverse events or cybersickness, confirming the initial feasibility of immersive VR in this population. Acceptance of the application was common during the execution of the three sessions, since all three participants finished the sessions without safety issues, problems with cybersickness, or other adverse events. The mean VRNQ-DE scores exceeded the threshold, especially for video and sound quality, proving the participants’ acceptance of and satisfaction with the immersive VR content.

The participants walked up to 1.3 km within their sessions, which averaged 28 min, and up to 1800 steps, demonstrating the quality of the proposed prototype not only for neurocognitive stimulation but also for combination with physical exercise within a safe environment. Anecdotal reports suggest emotional involvement and potential autobiographical memory activation. The software–hardware combination proved to be highly versatile; the setup was applied in different environments, demonstrating its portability and minimal infrastructure requirements. All participants were able, despite zero prior experience with gaming or virtual reality, to navigate the immersive VR. Minimal explanation and setup times facilitate the application’s use in care settings or home environments.

### 5.2. Comparison to Prior Work

We seek to extend the digital approach in cognitive stimulation research to immersive, open-world VR. We demonstrate the potential of state-of-the-art VR to expand therapeutic training programs. Unlike serious games, the software was modified to emphasise free exploration, provide immersiveness, and stimulate intrinsic motivation and self-agency. This approach is contrary to the goal- or score-driven approaches of other software in the field. Compared to earlier studies, which reported VR sickness, the use of modern hardware eliminated potential adverse effects during the use of VR.

### 5.3. Future Outlook

We propose a controlled multi-centre trial, including a control group, to compare our method against traditional CST methods and validate the NeuroVRX approach beyond feasibility. To obtain statistical evidence, we need to increase the sample size to be able to generalise the findings and gather data across gender and age for comparison with traditional CST methods. Trial protocols can further be extended to long-term exposure to assess the effects on cognitive abilities and to detect mood changes. In the mid to long term, it is possible to incorporate biometric monitoring (e.g., heart rate variability, pulse, gaze analysis, skin conduction) to quantify stress-related and emotional responses during training. Due to the fact that all participants were male, we want to highlight the importance of exploring gender differences while establishing a new form of CST. Furthermore, we plan to enable interaction with other players and socially intelligent non-player characters during the session. The goal is to create meaningful encounters within the VR.

Our strategic approach is to establish virtual CST programs as a component in CST training for clinics, in care facilities, and in home care settings. Further, we seek to investigate the inclusion of immersive digital VR in national dementia care reimbursement programmes, along with guidelines for its safe deployment in nursing homes and home care settings.

## Figures and Tables

**Table 1:** Inclusion and exclusion criteria (with International Classification of Diseases, 10th Revision (ICD-10) codes).

Inclusion Criteria	Exclusion Criteria
Existing Diagnosis early ADRD (G30)	Diagnosed epilepsy (G.40)
Competence to consent	diagnosed anxiety disorders (F40–F41)
	Impaired hearing (H90)
	Impaired vision (H54)
	Advanced dementia

**Table 2:** Session data in the forest scenario: duration (min), distance (km), steps for each session, and mean ± SD across participants.

	Participant A	Participant B	Participant C	Mean ± SD
Session duration (min)	21	34	30	28.3 ± 6.7
Session distance (km)	0.2	1.1	1.3	0.9 ± 0.6
Session steps	588	1807	1236	1210 ± 610

**Table 3:** Virtual Reality Neuropsychological Questionnaire—German version (VRNQ-DE).

	Participant A	Participant B	Participant C	Mean
User Experience	24	22	29	25.00
Immersion	4	3	5	4.00
Pleasantness	4	3	5	4.00
Graphics	6	6	7	6.33
Sound	6	4	5	5.00
VR Tech	4	6	7	5.67
Game Mechanics	20	25	18	21.00
Orientation	4	6	3	4.33
Movement	4	6	6	5.33
Safety	6	7	7	6.67
Exploration	6	6	2	5.67
VRNQ-DE Score	44	47	47	46.00
VRNQ-DE Threshold	45	45	45	45.00

**Table 4:** Change in mood scores (ASTS Post–Pre).

	Participant A	Participant B	Participant C
Trauer (Sadness)	0	0	0
Hoffnungslosigkeit (Hopelessness)	0	0	−1
Müdigkeit (Fatigue)	0	−1	+9
Positive Stimmung (Positive Mood)	+3	0	−9

## Data Availability

Registration and protocol of this study are available at the OSF (Open Science Framework) with the ID r6up9 (https://osf.io/r6up9, accessed on 20 November 2025). Questionnaire data, VD profile settings and questionnaires are available via the OSF.

## Abbreviations

The following abbreviations are used in this manuscript:

3D	Three-dimensional
ADRD	Alzheimer’s disease and related dementias
BMI	Body mass index
CST	Cognitive Stimulation Therapy
CSQ	Cyber Sickness Questionnaire
CSQ-VR	Cyber Sickness Questionnaire (VR)
GBD	Global Burden of Disease
GDPR	General Data Protection Regulation
NeuroVRX	Neurocognitive virtual reality exploration
POMS	Profile of Mood States
PwD	People with dementia
VR	Virtual reality
VRNQ	Virtual Reality Neuroscience Questionnaire
VRNQ-DE	Virtual Reality Neuroscience Questionnaire, German version
WHO	World Health Organization
Wi-Fi	Wireless local area network

## Appendix A

**Table A1:** Hardware configuration.

Item	Model / Configuration	Manufacturer
HMD	Meta Quest 3 (128 GB)	Meta Platforms, Inc. (Menlo Park, CA, USA)
Controllers	Meta 3 Touch Plus; gesture and hand tracking off	Meta Platforms, Inc. (Menlo Park, CA, USA)
Host Machine	MSI Titan 18 HX A14VIG; Intel(R) Core(TM) i9-14900HX 2.20 GHz; 128 GB RAM; RTX 4090 Laptop GPU; 32 GB RAM	Micro-Star International Co., Ltd. (MSI) (Zhonghe District, New Taipei City, Taiwan)
OS & Drivers	Windows 11 Pro 24H2 (26,100.3476); NVIDIA 31.0.15.5222 (11 April 2024)	Microsoft Corporation (Redmond, WA, USA); NVIDIA Corporation (Santa Clara, CA, USA)
Audio I/O	HMD integrated speakers; system volume 75%	—

**Table A2:** Networking and streaming.

Link	Wi-Fi 5 GHz: 1300 Mbps (802.11ac), WPA2, dedicated SSID
Router/AP	TP-Link (Shenzhen, Guangdong, China) Archer C7 AC1750 v2, FW: Archer C7(EU) V2 241108
Codec/Transport	Virtual Desktop; 120 FPS; HEVC; SSW Enabled; Snapdragon Game Super Resolution; Video Buffering

**Table A3:** Software runtime and settings.

Component	Software	Version/ Notes	Developer/Publisher
Application	The Elder Scrolls V: Skyrim VR	1.4.15	Bethesda Softworks LLC (Rockville, MD, USA)
	Mad God Overhaul	2.6	by Moyse06 (https://www.nexusmods.com/skyrimspecialedition/mods/107780, accessed on 27 November 2025)
	Mod Organizer 2	2.5.0	Mod Organizer 2 Team (https://github.com/ModOrganizer2, accessed on 27 November 2025)
VR runtime	Steam	build 1747701111	Valve Corporation (Bellevue, WA, USA)
	SteamVR	2.11.2	Valve Corporation (Bellevue, WA, USA)
	Virtual Desktop	1.34.8-72	Virtual Desktop, Inc.; (Los Angeles, CA, USA)
	Natural Locomotion	build 15890947 (Oct 2024)	myousoftware (https://store.steampowered.com/publisher/myou, accessed on 27 November 2025)

**Table A4:** Overview of variables.

Variable	Description
Subject	Identifier for each participant
Gender	Gender of the participant
Age of Subject	Age of the participant at the time of the study
Age of Diagnosis	Age at which the participant received their diagnosis
Prone to Motion Sickness	Whether the participant is prone to motion sickness
Gaming Experience	Level of previous gaming experience
VR Experience	Level of previous virtual reality experience
Session Scenario	Name of the VR session scenario
Session Duration (minutes)	Duration of the VR session
Session Distance (km)	Distance covered during the VR session
Session Steps	Number of steps performed during the VR session
Recommendation	If a participant would recommend the experience
ASTS-pre and ASTS-post [29]	Profile of Mood States (German POMS)
Sadness	Sadness score
Hopelessness	Hopelessness score
Fatigue	Fatigue score
Positive Mood	Positive mood score
VRNQ-DE [31]	VR Neuropsychological Questionnaire (German)
User Experience	User experience score
Gameplay Mechanics	Gameplay mechanics score
CSQ [30]	Measures of cybersickness (German)
Nausea	Nausea score
Balance	Balance disturbance score
Eye Strain	Eye strain score

**Table A5:** Profile of Mood States-German (ASTS) scores before and after VR session.

	Participant A	Participant B	Participant C	$\bar{x}$
ASTS-pre				
Sadness	3	3	3	3.00
Hopelessness	3	3	4	3.33
Fatigue	4	8	4	5.33
Positive Mood	21	12	26	19.67
ASTS-post				
Sadness	3	3	3	3.00
Hopelessness	3	3	3	3.00
Fatigue	4	7	13	8.00
Positive Mood	24	12	17	17.67

**Table A6:** Cyber Sickness Questionnaire (CSQ-DE) results.

Cyber Sickness	Participant A	Participant B	Participant C	$\bar{x}$
Nausea	2	2	2	2
Balance	2	2	2	2
Eye Strain	2	2	2	2
Total	6	6	6	6
